# Characteristics of Tailored Text Messages Associated With Increased Physical Activity Among Cardiac Rehabilitation Enrollees: Secondary Analysis of a Microrandomized Trial

**DOI:** 10.2196/79792

**Published:** 2026-08-11

**Authors:** Namratha Atluri, Kashvi Gupta, Tanima Basu, Evan Luff, Jieru Shi, Thomas Boyden, Bhramar Mukherjee, Sachin Kheterpal, Predrag Klasnja, Walter Dempsey, Brahmajee K Nallamothu, Jessica R Golbus

**Affiliations:** 1 Department of Internal Medicine University of Michigan Ann Arbor, MI United States; 2 Division of Nephrology Department of Internal Medicine University of California, San Francisco San Francisco, CA United States; 3 Division of Cardiovascular Diseases Department of Internal Medicine University of Michigan Ann Arbor, MI United States; 4 Statistical Laboratory Department of Pure Mathematics and Mathematical Statistics University of Cambridge Cambridge United Kingdom; 5 Division of Cardiovascular Diseases Department of Internal Medicine Corewell Health Grand Rapids, MI United States; 6 Department of Biostatistics Yale School of Public Health New Haven, CT United States; 7 Department of Anesthesiology University of Michigan Ann Arbor, MI United States; 8 School of Information University of Michigan Ann Arbor, MI United States; 9 Department of Biostatistics School of Public Health University of Michigan Ann Arbor, MI United States

**Keywords:** cardiac rehabilitation, mobile health, mHealth, physical activity, text messages, tailoring, personalization

## Abstract

**Background:**

Emerging data suggest that text message–based mobile health interventions may enhance physical activity levels in patients with cardiovascular disease enrolled in cardiac rehabilitation. The optimal characteristics of texts that lead to maximal patient engagement and drive meaningful behavioral change are not well understood.

**Objective:**

This study aimed to understand how text- and participant-level characteristics impact physical activity levels after text delivery.

**Methods:**

The VALENTINE (Virtual Application-Supported Environment to Increase Exercise) study was a randomized controlled trial designed to evaluate a mobile health intervention delivered to low- and moderate-risk adults enrolled in cardiac rehabilitation. Embedded within this study was a microrandomized trial focused on the effect of texts on physical activity levels among intervention participants. Participants in the intervention group received texts through a smartwatch (Apple Watch or Fitbit Versa) that were tailored to the time of day, day of the week (weekday vs weekend), weather, and time since enrollment in cardiac rehabilitation. Texts also differed in content type (walking vs antisedentary) and in the level of personalization (inclusion of the participant’s name or not). Delivery was randomized at 4 user-selected time points daily, with participants having a 25% probability of receiving a text at any time point. The primary outcome was step count 60 minutes after a decision point. This analysis focuses on the text- and participant-level factors that moderated the intervention’s effect on the primary outcome. Given potential measurement differences determined a priori, analyses were stratified by device type and phase of cardiac rehabilitation and adjusted for age, sex, and baseline activity status using a generalization of regression analysis.

**Results:**

More than 70,552 randomizations occurred in 108 participants (mean age 59.5, SD 10.7 years; n=36, 33.3% female; n=19, 17.6% non-White; n=68, 63% Apple Watch users) over 6 months. Overall, no text characteristics (including personalization with the participant’s name) or participant characteristics (including baseline physical activity) consistently impacted text responsiveness for either device type. Although the findings were not consistently significant between device types and across phases of the trial, there was a trend toward increased responsiveness to texts that promoted walking (compared to antisedentary texts) and that were delivered to younger (aged <65 years) and male participants.

**Conclusions:**

In this randomized clinical trial, we found that tailored texts improved physical activity levels among cardiac rehabilitation enrollees in the initiation phase, but this effect was not explained by text- or participant-level moderators. Additional work is needed to explore the impact of tailoring based on an extended set of personal and environmental factors to optimize the delivery and efficacy of text message–based interventions.

**Trial Registration:**

ClinicalTrials.gov NCT04587882; https://clinicaltrials.gov/study/NCT04587882

**International Registered Report Identifier (IRRID):**

RR2-10.1016/j.ahj.2022.02.012

## Introduction

Center-based cardiac rehabilitation (CR) is an evidence-based lifestyle program that improves functional capacity and quality of life and reduces morbidity and mortality in patients with cardiovascular disease (CVD) [1-5]. Despite these well-established benefits, participation in CR remains low, and physical activity levels often decline over time after program completion [3-5]. Emerging data suggest that mobile health (mHealth) interventions may be effective in extending the benefits of center-based CR beyond the supervised setting [6-10]. mHealth interventions encompass a range of technologies, including text messages, mobile phone apps, and transmission of physiological data (eg, heart rate, step count, and blood pressure) from sensors to mobile devices and web-based platforms [9]. Among these, texts are the most widely accessible. To drive meaningful and sustainable long-term behavioral change, optimizing key text features is crucial. Through strategic timing and frequency of delivery, personalized content, and engaging user interfaces, tailored text-based interventions can promote patient engagement and usability while preventing habituation to improve clinical outcomes [9,10].

The VALENTINE (Virtual Application-Supported Environment to Increase Exercise) study evaluated the impact of an mHealth intervention combining texts, a mobile app, and a smartwatch (Apple Watch or Fitbit Versa) on physical activity levels in enrollees undergoing CR over a 6-month period [11-13]. Nested within the VALENTINE study was a microrandomized trial designed to study the effect of texts on physical activity levels throughout the day. Microrandomized trials are a novel experimental design in which interventions such as texts are repeatedly randomized for delivery at different time points throughout the day. This powerful design enables assessment of the causal effects of individual intervention components on near-term outcomes, such as step count following text delivery, which are thought to influence longer-term behavioral or health outcomes, including sustained physical activity. The primary analysis of the VALENTINE study’s microrandomized trial showed that the 60-minute step count increased early after text delivery for both Apple Watch and Fitbit users, with differential results by device type thereafter [12]. Understanding which text characteristics and contextual factors promoted this increase in physical activity, at least initially, is necessary to develop more effective text interventions.

Herein, we present a prespecified secondary analysis from the VALENTINE study’s microrandomized trial, evaluating the impact of text tailoring on physical activity levels. The trial leveraged real-time data from participants’ smartwatches about their current environment to tailor texts with the goal of promoting low-level physical activity. In this analysis, the primary objective was to evaluate the impact of both participant-level factors and text characteristics hypothesized to moderate intervention efficacy. We hypothesized that the step count in the 60 minutes following text delivery would differ significantly based on text characteristics, such as time of delivery, personalization with the participant’s name, and level of activity prior to text delivery, as well as participant-level factors such as age, sex, and baseline activity status.

## Methods

### Overview

The VALENTINE study was a prospective, multicenter randomized clinical trial that remotely delivered an mHealth intervention to low- and moderate-risk patients enrolled in center-based CR (ClinicalTrials.gov NCT04587882). The primary objectives of the overall trial were (1) to evaluate the efficacy of a multicomponent mHealth intervention for augmenting and extending the benefits of center-based CR and (2) to evaluate the impact of text message delivery specifically on physical activity levels. The methodological details of the study have been previously described [11-13]. Briefly, adults aged ≤75 years who had completed 2 CR visits based on a qualifying diagnosis, owned a compatible smartphone, and spoke English were randomized to the intervention or control arm of the study. Patients who were unsafe to exercise without supervision due to a high-risk cardiovascular condition were excluded.

### Ethical Considerations

This study was approved by the institutional review board of the University of Michigan (HUM00162365). Informed consent was obtained from all participants before randomization. Efforts were made to preserve patient privacy whenever possible, including instructions on removing the mobile app from their phones at the end of the study to prevent passive data collection. Other than wearable devices, no other compensation was provided for study participation.

### Study Procedures

The VALENTINE study launched in October 2020 at an academic medical center and in July 2021 at a large community health care center in Michigan. Both the intervention and control groups underwent remote consent and enrollment visits and received a wearable device compatible with their smartphone (Apple Watch Series 4; Apple Inc or Fitbit Versa 2; Fitbit Inc) to track daily physical activity and receive texts. Additionally, participants in both groups participated in center-based CR concurrently.

### Description of the Intervention

**Figure 1:**
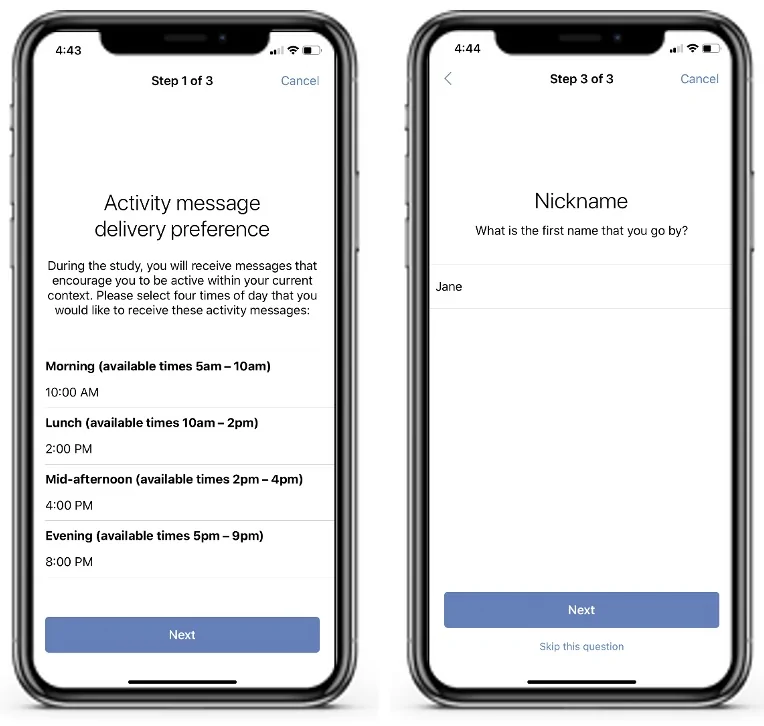
Screenshots from the VALENTINE (Virtual Application-Supported Environment to Increase Exercise) mobile app showing participant selection of preferred text messages delivery times and entry of a nickname for text personalization during enrollment.

**Figure 2:**
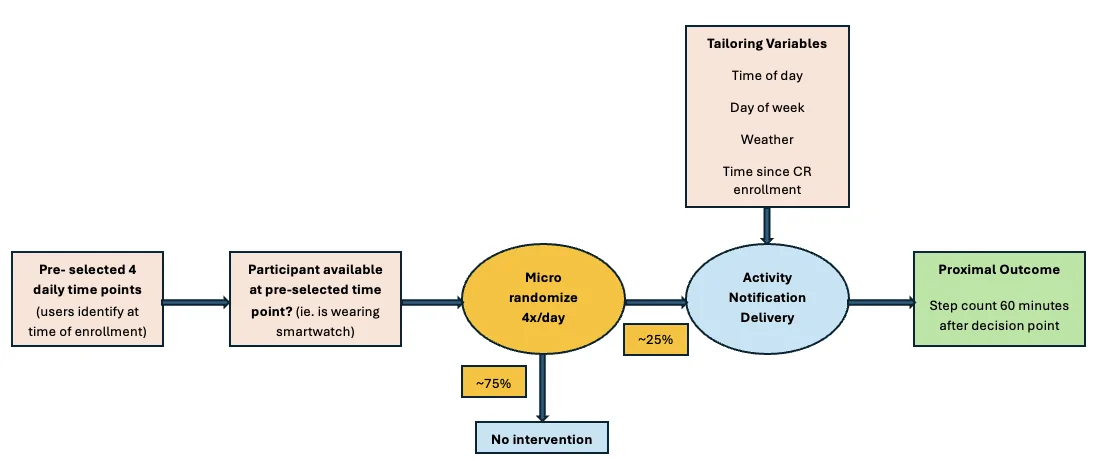
Microrandomized trial design diagram. CR: cardiac rehabilitation.

The VALENTINE study was designed to augment and extend the benefits of CR through an mHealth intervention. The intervention consisted of a mobile app that allowed activity tracking, goal setting, and microrandomized texts (Figure 1). The texts were delivered by SMS to participants’ smartwatches and phones but could also be reviewed in the mobile study app. Texts were tailored to the time of day (morning, lunchtime, afternoon, or evening), day of the week (weekday vs weekend), weather (ie, indoor-based texts delivered if snow or rain was predicted), and time since enrollment in CR (corresponding to the initiation, 0-30 days; maintenance, 31-120 days; and completion, 121-182 days phases of CR). Participants were randomized to receive a text or no text at 4 user-selected time points (ie, decision points), with texts selected at random based on participants’ environments (Figure 2). Participants had a 25% probability of receiving a text at each time point.

**Figure 3:**
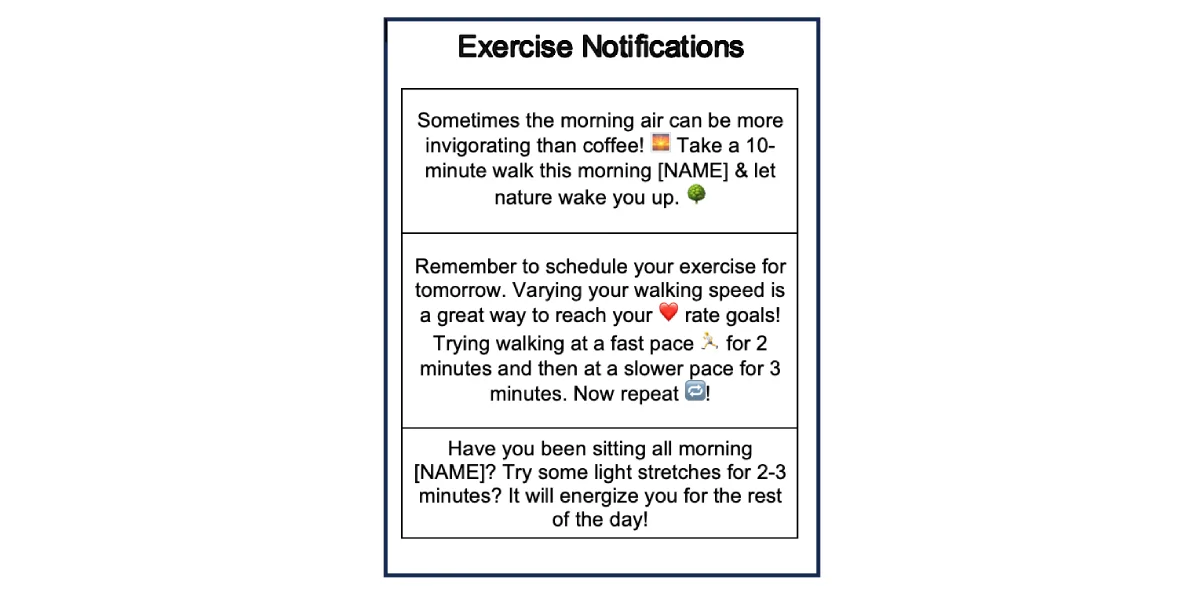
Example of activity text messages used in the VALENTINE (Virtual Application-Supported Environment to Increase Exercise) study.

This secondary analysis focuses on the results of activity texts. Activity texts were designed to encourage low levels of physical activity in the hour after delivery and were written to be low intensity, short duration, and actionable in real time, as opposed to exercise, which is a planned behavior and often longer in duration and higher in intensity (Figure 3). There were a total of 284 possible activity texts that varied in recommended action (encouraging a short walk vs standing up and stretching) and framing (emphasizing the benefits of being active vs highlighting missed opportunities to meet goals). Approximately 50% of the activity texts were personalized with participants’ preferred names, and approximately 30% included emojis or hyperlinks to the mobile app. The effectiveness of texts was assessed through proximal outcomes, which refer to the near-term effects of the intervention. The primary proximal outcome for activity texts was the step count 60 minutes following a decision point.

### Statistical Analysis

Baseline characteristics are described as mean (SD) for continuous variables and counts (percentage) for categorical variables. Student *t* tests (2-tailed) or chi-square tests were used for bivariate comparisons of continuous and categorical variables, respectively.

We decided a priori to analyze the data by device type (Apple Watch vs Fitbit) due to known differences in how each device measures physical activity [14]. The study was divided into initiation (days 1-30), maintenance (days 31-120), and completion (days 121-182) phases to account for participants’ progression through CR. On the basis of the primary analysis results, which demonstrated an early effect of texts for both device types, this analysis focused on the overall and initiation phases of the study [12].

Our primary outcome for this analysis was the step count in the 60 minutes following a decision point. As the step count was positively skewed, 0.5 was added to all step counts before log transformation. All analyses were performed using a centered and weighted least squares method, a standard method to assess time-varying causal effects of a digital intervention in a microrandomized trial [15]. This method estimates parameters in models for treatment effects and allows the robust inclusion of covariates to reduce noise. Similar to the generalized estimating equations and multilevel models, this method accommodates the nested nature of the data and within-participant correlation across time and accounts for sequential randomization to estimate causal treatment effects. Additionally, regardless of sample size, this method yields consistent and asymptotically normal estimates of the marginal or moderated treatment effect of interest. We assessed causal effect moderation by the following covariates: age (<65 vs >65 years), baseline physical activity (>7000 vs <7000 steps in the first week of the study), sex, and step count 30 minutes before each decision point. Device-specific models evaluated the effect of delivering a text compared with no text at each decision point. At each decision point, participants were considered available to receive the intervention if they were wearing their smartwatches, which was operationalized by requiring at least 1 heart rate measurement on participants’ smartwatches at least 30 minutes prior to a decision point. The interaction between treatment and a series of contextual factors (time of text delivery, weekend vs weekday, text content [antisedentary vs activity promoting], and personalization of the text using the participant’s name), as well as participant characteristics (age category, sex, baseline activity status, presence of heart failure, and indication for CR referral), was explored. A *P* value <.05 was used to establish significance. All analyses were performed using SAS (version 14.2; SAS Institute Inc).

## Results

### Overview

**Figure 4:**
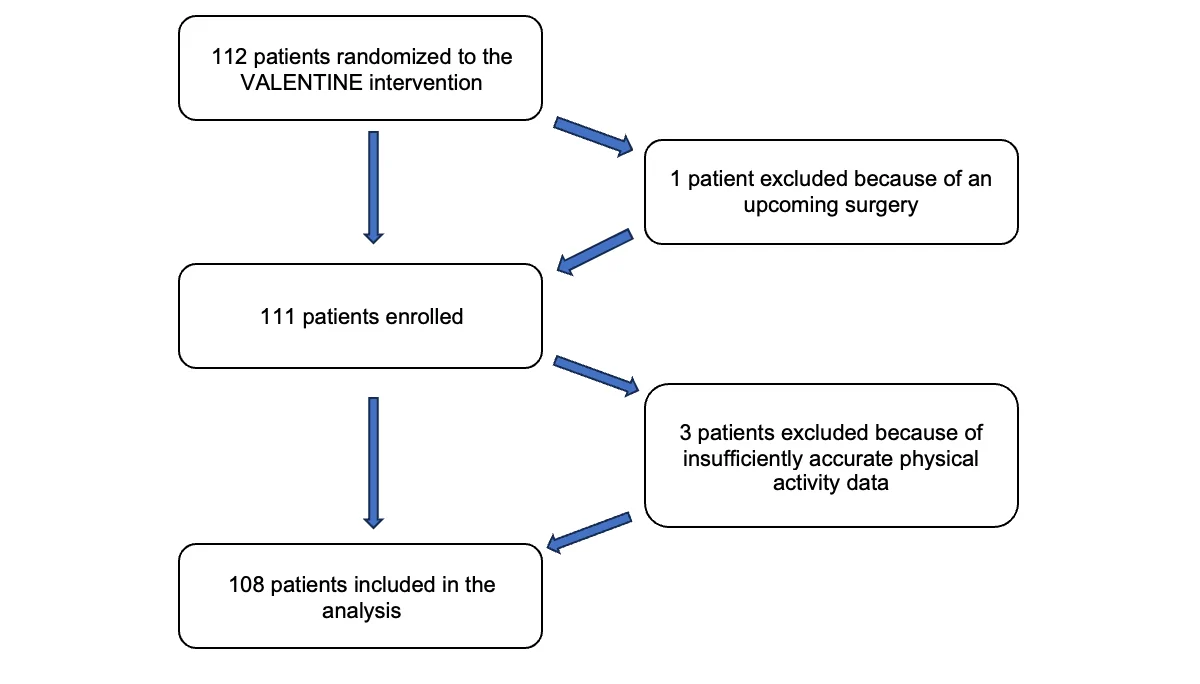
CONSORT (Consolidated Standards of Reporting Trials) flow diagram. VALENTINE: Virtual Application-Supported Environment to Increase Exercise.

From October 23, 2020, to March 25, 2022, 112 participants were randomized to the intervention arm of the study. Only 1 participant was excluded after randomization but before enrollment due to failure to meet the inclusion criteria (ie, upcoming surgery), and 3 had incomplete physical activity data for analysis (Figure 4). An additional 2 participants withdrew from the study after enrollment but allowed data collected up until their withdrawal date to be used. Of the 108 remaining participants, 36 (33.3%) were female, 89 (82.4%) were White, and 101 (93.5%) were non-Hispanic. Participants’ mean age was 59.5 (SD 10.7) years, and their mean BMI was 30.4 (SD 6.5) kg/m^2^; 68 (63%) participants owned an iPhone and were provided with an Apple Watch (Table 1). Throughout the study, these participants underwent 70,552 randomizations to receive or not receive a text over a median of 180.5 (IQR 172.5-182.0) days. Participants were available for 75.9% of decision points (77.3% Apple Watch vs 73.4% Fitbit), with availability defined as having at least 1 smartwatch heart rate measurement in the 30 minutes prior to randomization. On average, 0.91 (SD 0.86) texts per day were sent, although participants were only available to receive 0.80 texts per day.

**Table 1:** Baseline characteristics of participants enrolled in the intervention arm of the VALENTINE (Virtual Application-Supported Environment to Increase Exercise) study.

Characteristics			Overall (N=108)		Fitbit (n=40)		Apple Watch (n=68)
Age (years), mean (SD)			59.5 (10.7)		58.3 (9.6)		59.6 (11.4)
**Sex, n (%)**							
	Male	72 (66.7)		26 (65)		46 (67.6)	
	Female	36 (33.3)		14 (35)		22 (32.4)	
**Race, n (%)**							
	Asian	6 (5.6)		1 (2.5)		5 (7.4)	
	Black	4 (3.7)		4 (10)		0 (0)	
	Other or unknown	9 (8.3)		5 (12.5)		4 (5.9)	
	White	89 (82.4)		30 (75)		59 (86.8)	
**Ethnicity, n (%)**							
	Hispanic	2 (1.9)		1 (2.5)		1 (1.5)	
	Non-Hispanic	101 (93.5)		39 (97.5)		62 (91.2)	
	Unknown	5 (4.6)		0 (0)		5 (7.4)	
**Study site, n (%)**							
	Michigan Medicine	89 (82.4)		34 (85)		55 (80.9)	
	Spectrum Health	19 (17.6)		6 (15)		13 (19.1)	
**Indication for cardiac rehabilitation, n (%)**							
	PCI^a^	58 (20.4)		22 (55)		36 (52.9)	
	CABG^b^	19 (17.6)		9 (22.5)		10 (14.7)	
	Valve repair or replacement	22 (20.4)		7 (17.5)		15 (22.1)	
	PCI or CABG and valve repair or replacement	2 (1.9)		0 (0)		2 (2.9)	
	CAD^c^ or acute coronary syndrome, not revascularized	7 (6.5)		2 (5)		5 (7.4)	
BMI^d^ (kg/m^2^), mean (SD)			30.4 (6.5)		31.4 (7.2)		29.8 (6.0)
**Comorbidities, n (%)**							
	Atrial fibrillation or flutter^d^	29 (26.9)		8 (20)		21 (30.9)	
	CAD or myocardial infarction	89 (82.4)		35 (87.5)		54 (79.4)	
	Diabetes mellitus^d^	30 (27.8)		17 (42.5)		13 (19.1)	
	Hypertension^d^	73 (67.6)		29 (72.5)		44 (64.7)	
	Heart failure	20 (18.5)		7 (17.5)		13 (19.1)	
	History of valve repair or replacement	28 (25.9)		9 (22.5)		19 (28.9)	

^a^PCI: percutaneous coronary intervention.

^b^CABG: coronary artery bypass grafting.

^c^CAD: coronary artery disease.

^d^Data available for Michigan Medicine patients only.

### Impact of Texts on Apple Watch Users

Baseline mean daily step count was 7536.0 (SD 3666.3) steps for Apple Watch users. During the initiation period of the study, delivering a text increased step count by a nonsignificant 10% (95% CI −1% to 20%; *P*=.05) in the 60 minutes after text delivery. Over the entire study period, walking texts increased step count more than antisedentary texts, although the difference was nonsignificant, with a relative risk ratio (RRR) of 1.08 (95% CI 1.00-1.17; *P*=.05; Table 2). In the study overall and in the initiation phase, text tailoring based on time of day, day of the week (weekend vs weekday), or personalization with participants’ names did not significantly change the impact of texts on physical activity levels.

**Table 2:** Moderator analysis of contextually tailored text messages to promote physical activity among Apple Watch users in the VALENTINE (Virtual Application-Supported Environment to Increase Exercise) study.

Moderators			Overall							Initiation phase				
			RRR^a^		95% CI		*P* value		RRR			95% CI		*P* value
**Time of day**														
	Morning vs lunch	0.93		0.84-1.02		.14		0.87			0.63-1.21		.42	
	Morning vs afternoon	0.95		0.85-1.06		.36		0.97			0.72-1.29		.81	
	Morning vs evening	0.93		0.84-1.03		.20		0.86			0.63-1.19		.37	
	Lunch vs afternoon	1.02		0.93-1.12		.65		1.10			0.86-1.42		.44	
	Lunch vs evening	1.00		0.91-1.10		.99		0.99			0.79-1.24		.92	
	Afternoon vs evening	0.98		0.90-1.06		.60		0.89			0.70-1.14		.36	
Time of week: weekend vs weekday			0.97		0.90-1.05		.45		0.96			0.78-1.18		.70
Text type: walking vs antisedentary			1.08		1.00-1.17		.05		1.05			0.84-1.31		.67
Personalization: name vs no name			0.99		0.93-1.04		.58		0.88			0.75-1.03		.11
Age category: young (<65 years) vs old (>65 years)			1.04		0.97-1.11		.23		*1.27* ^b^			*1.07-1.51*		*.01*
Sex: male vs female			0.99		0.93-1.05		.72		*1.27*			*1.05-1.52*		*.01*
Activity status: less active vs active			0.99		0.93-1.05		.64		0.91			0.76-1.10		.35
Heart failure: present vs absent			1.03		0.97-1.10		.31		1.02			0.81-1.30		.85
**Indication for cardiac rehabilitation**														
	PCI^c^ vs CABG^d^	1.04		0.95-1.14		.39		0.84			0.56-1.26		.40	
	Valve repair or replacement vs CABG	1.03		0.93-1.15		.56		0.72			0.47-1.09		.12	
	Valve repair or replacement vs PCI	0.99		0.91-1.08		.85		0.85			0.70-1.05		.13	

^a^RRR: relative risk ratio.

^b^Italicized values indicate statistical significance.

^c^PCI: percutaneous coronary intervention.

^d^CABG: coronary artery bypass graft.

When analyzing the impact of texts based on participant characteristics, younger participants (RRR 1.27; 95% CI 1.07-1.51; *P*=.006; Table 2) and male participants (RRR 1.27; 95% CI 1.05-1.52; *P*=.01; Table 2) were more responsive to texts than older participants (age >65 years) and female participants during the initiation phase of the study. The indication for CR, the presence or absence of heart failure, and baseline physical activity levels did not significantly impact responsiveness to texts.

### Impact of Texts on Fitbit Users

Baseline mean daily step count was 7011.2 (SD 3711.5) steps for Fitbit users. In the initiation phase of the study, texts increased step counts by 17% (95% CI 7%-28%; *P*<.001) in the 60 minutes after text delivery. In the initiation phase of the study, there was a trend, although not statistically significant, toward increased step count when texts were delivered at lunch compared to the afternoon (RRR 1.35; 95% CI 1.00-1.82; *P*=.05; Table 3). This trend, although not statistically significant, persisted for the overall study period (RRR 1.11; 95% CI 0.99-1.24; *P*=.07; Table 3). In the study overall and in the initiation phase, text tailoring based on day of the week or personalization with participants’ names did not significantly impact step count.

**Table 3:** Moderator analysis of contextually tailored text messages to promote physical activity among Fitbit Versa 2 users in VALENTINE (Virtual Application-Supported Environment to Increase Exercise) study.

Moderators		Overall				Initiation phase		
		RRR^a^	95% CI	*P* value	RRR		95% CI	*P* value
**Time of day**								
	Morning vs lunch	0.94	0.82-1.07	.34	1.02		0.72-1.44	.91
	Morning vs afternoon	1.04	0.91-1.19	.60	1.38		0.96-1.97	.08
	Morning vs evening	1.04	0.89-1.22	.61	1.18		0.82-1.69	.38
	Lunch vs afternoon	1.11	0.99-1.24	.07	1.35		1.00-1.82	.05
	Lunch vs evening	1.11	0.97-1.28	.13	1.15		0.82-1.63	.42
	Afternoon vs evening	1.00	0.89-1.14	.95	0.85		0.59-1.24	.41
Time of week: weekend vs weekday		0.95	0.87-1.03	.21	0.94		0.70-1.26	.67
Text type: walking vs antisedentary		1.00	0.92-1.08	.95	0.93		0.73-1.20	.60
Personalization: name vs no name		0.99	0.93-1.06	.87	0.89		0.71-1.12	.31
Age category: young (<65 years) vs old (>65 years)		0.98	0.91-1.07	.70	0.94		0.78-1.13	.52
Sex: male vs female		*1.10* ^b^	*1.02-1.18*	*.01*	1.04		0.88-1.23	.68
Activity status: less active vs active		0.95	0.88-1.02	.14	0.88		0.75-1.04	.15
Heart failure: present vs absent		0.99	0.89-1.11	.92	0.91		0.92-1.30	.31
**Indication for cardiac rehabilitation**								
	PCI^c^ vs CABG^d^	0.99	0.93-1.06	.79	0.88		0.74-1.06	.18
	Valve repair or replacement vs CABG	1.01	0.92-1.11	.89	*1.31*		*1.03-1.67*	*.03*
	Valve repair or replacement vs PCI	1.02	0.92-1.12	.76	*1.48*		*1.17-1.87*	*.001*

^a^RRR: relative risk ratio.

^b^Italicized values indicate statistical significance.

^c^PCI: percutaneous coronary intervention.

^d^CABG: coronary artery bypass graft.

When analyzing the impact of texts based on participant characteristics, male participants were more responsive to texts than female participants (RRR 1.10; 95% CI 1.02-1.18; *P*=.01; Table 3) in the overall study. The indication for CR significantly impacted participants’ responsiveness to texts, with participants who underwent valve repair or replacement being more responsive to texts than those who underwent percutaneous coronary intervention (RRR 1.31; 95% CI 1.03-1.67; *P*=.03; Table 3) or coronary artery bypass grafting (RRR 1.48; 95% CI 1.17-1.87; *P*=.001; Table 3). Sex category, the presence or absence of heart failure, and baseline physical activity status did not significantly impact responsiveness to texts.

## Discussion

### Principal Findings

In the VALENTINE mHealth intervention designed to augment the benefits of CR through texts, we found that texts increased step counts in the 60 minutes following delivery by a statistically significant 17% for Fitbit users and a nonsignificant 10% for Apple Watch users in the initiation phase of the study. In this secondary analysis, the primary objective was to evaluate the intervention-level and participant-level moderators of text efficacy, examining the study period overall as well as the initiation phase, during which texts were most effective. We found that contextual factors related to texts, such as timing of delivery and content of texts, did not consistently affect step count in either Apple Watch or Fitbit users. Notably, although personalization of texts with participants’ names was hypothesized to increase responsiveness based on prior studies [16-18], it did not result in increased step count compared to nonpersonalized texts for either device type in our study. Although participant-level factors, such as sex and age, occasionally had significant effects, these were not consistent across device types and phases of the trial. Notably, baseline physical activity was predicted to be a moderator of text responsiveness (participants with high baseline activity may not find texts as effective), but there was no statistically significant difference in text responsiveness between those with low and high baseline activity for either device type.

This study adds to a growing body of literature exploring the efficacy of text-based mHealth interventions and specifically ways to optimize delivery to elicit behavioral change. Prior studies have shown that texts can motivate patients with CVD to augment physical activity in the form of increased step count and time spent engaging in moderate-intensity exercise [16-23]. Meta-analyses, mostly in patient populations without CVD, have also shown that personalized and tailored texts that account for user preferences, baseline characteristics, or real-time contextual data are more effective than generic texts in promoting engagement and responsiveness [18-21]. Although previous studies have analyzed different forms of text personalization and framing separately, no study has directly compared and evaluated multiple types of text tailoring within the same participant population, especially in patients with CVD [17-23]. Our study sought to address this gap by using a novel microrandomized trial design to randomize patients with CVD to different types of tailored texts and determine which factors were most effective for promoting physical activity.

Although the VALENTINE study did not identify text attributes that consistently increased activity levels, these results should not necessarily be interpreted as evidence that tailoring is ineffective but rather as a reflection of the complexity involved in tailoring texts. Despite focused efforts to tailor texts based on diverse factors hypothesized to be salient based on prior work [17,20,21,23], it is possible that the texts were not tailored to the most impactful factors, at least for this study population. For example, prior studies suggested that users desire personalized texts, which was enacted in the study by including participants’ names in a subset of texts [16,17]. However, it is possible that participants seek personalization in other ways not included in this study, such as in the context of their specific health goals, recent activity trends, or work schedules [21-23]. Additionally, given participant heterogeneity, it is likely that there are inherent differences between participants that may make the same tailored text effective for some but not for others. More sophisticated strategies, such as the use of reinforcement learning algorithms, may be needed to account for these individual-level differences so that participants receive the types of texts to which they have responded historically [24].

Despite the results lacking statistical significance, the VALENTINE study did reveal some interesting associations that may warrant further exploration. For example, the content of texts appeared to be influential, with texts that promoted walking leading to a higher step count than texts that promoted antisedentary activities among Apple Watch users. The timing of texts may also matter, with Fitbit users responding more often to texts delivered at lunch, when participants may have had a work break to engage in physical activity. These are less explored but vital text attributes that are hypothesis-generating and should serve as early evidence for future work.

Our study also found that results varied between Apple Watch and Fitbit users, although the smaller participant sample size and lack of statistically significant results limit interpretation. Prior research has shown that key differences between the Apple Watch and the Fitbit, such as user interface, functionality, integration into daily life, and activity data collection methodology, influence user engagement and data accuracy [11-14]. Studies have also suggested differences in the characteristics of individuals who gravitate toward 1 device type over the other, with the Fitbit being marketed primarily as a health and fitness tool while the Apple Watch is a multipurpose device. However, very few studies have directly compared how the 2 devices could mediate behavioral change in mHealth interventions and what customizable factors matter for each device type. Future studies should explore how intrinsic features of wearable devices may cater to different individuals and contexts and thereby influence the efficacy of mHealth interventions.

Our study has several strengths. First, our study used a novel microrandomized design, a powerful experimental design for making causal inferences [25]. Through sequential randomized assignment, we were able to assess time-varying and contextual factors with the potential to moderate intervention efficacy, an inherent limitation of randomized controlled trials, which can only assess the impact of an intervention package in aggregate. Second, we explored multiple layers of tailoring, including text content and participant-level factors. Few studies have similarly analyzed multiple moderators of texts or tailored texts to factors such as content type (eg, walking vs antisedentary). Third, with the primary outcome being step count 60 minutes after a decision point, our study assessed the immediate and likely direct impact of texts rather than a more distant outcome that could be explained by confounding factors or other intervention components, as many mHealth interventions have multiple intervention components. Finally, our study included both Apple Watch and Fitbit users, capturing comparative data on wearable devices in the context of mHealth interventions and increasing participant inclusivity.

Our study also has limitations. First, the sample size for individual moderator subgroups was small, potentially limiting our ability to detect differences by text type or participant-level factors. Thus, this analysis should be viewed as hypothesis-generating but not confirmatory. However, we did collect a rich dataset consisting of 70,552 serial randomizations, which allowed powerful assessments of causal effects. Second, although the texts were tailored to many factors, other potentially impactful factors, such as real-time activity data or user preferences regarding text characteristics, were not incorporated. Future iterations will focus on adding this layer of personalization. Third, although baseline characteristics, such as demographics, baseline activity status, and indication for CR, were evaluated as moderators, other participant-level factors relevant to an mHealth trial were not assessed, such as digital literacy. However, given the highly accessible delivery format of texts, digital literacy is less likely to affect their impact. Fourth, although texts were delivered at a time when participants were thought to be available, text engagement data were not collected (ie, whether and when participants read the text on their smartwatch or opened it in the mobile app). Therefore, step count assessment following text delivery may not have always captured activity change after viewing a text or accounted for the fact that a participant was already active at the time of viewing the text. Nonetheless, the availability data still suggest meaningful exposure to the intervention in a timely manner. Fifth, the sample contained only low and moderate-risk patients with CVD who already owned smartphones, were enrolled in center-based CR, and had relatively high activity at baseline, suggesting a population with higher levels of digital literacy and intrinsic motivation. Given these already optimized characteristics at baseline, there may have been a ceiling effect in terms of how much more benefit participants gained from tailored texts. However, our sample is diverse in other clinically meaningful ways (more than one-third of participants were aged ≥65 years, and a subset of participants were from a community setting); therefore, our findings are still applicable to real-world CR populations. Finally, although we compared the relative impact of different tailoring variables, the study evaluated a package of tailored texts compared to no text and did not compare the impact of tailored texts to that of generic texts. Thus, we are unable to draw conclusions regarding the impact of tailoring more generally.

### Conclusions

Overall, text message-based mHealth interventions are a promising approach for increasing physical activity levels among CR enrollees. In this secondary analysis of a microrandomized trial embedded within the VALENTINE study, we found that although tailored texts were associated with short-term increases in physical activity, no individual text-level or participant-level characteristic consistently moderated responsiveness across time periods and device types. These findings indicate that commonly used tailoring strategies, such as timing of text delivery, content type, or personalization with a participant’s name, are insufficient on their own to optimize engagement and behavioral response in a heterogeneous patient population. Effective text tailoring likely requires a broader and more dynamic set of variables, including real-time behavioral data, evolving user preferences, and device-specific contexts. Future research should focus on developing learning algorithms that can learn and adapt to user preferences and adjust text delivery over time to more effectively augment and extend the benefits of center-based CR.

## Data Availability

The data that support the findings of this study are available from the corresponding author upon reasonable request.

## Abbreviations

CR: cardiac rehabilitation
CVD: cardiovascular disease
mHealth: mobile health
RRR: relative risk ratio
VALENTINE: Virtual Application-Supported Environment to Increase Exercise
